# Anesthetic technique is associated with complication risk and healthcare utilization following total shoulder arthroplasty

**DOI:** 10.1007/s00590-026-04990-w

**Published:** 2026-10-21

**Authors:** Tarek Haj Shehadeh, Hannah Dieterle, Jawaad Chaudhry, Gary Updegrove, Nathan Lanham

**Affiliations:** Penn State Milton S. Hershey Medical Center, Hershey, USA

**Keywords:** Shoulder replacement, Regional anesthesia, General anesthesia, Complications

## Abstract

**Purpose:**

Compare perioperative complications and healthcare utilization among total shoulder arthroplasty (TSA) patients receiving general (GA), regional (RA), or combined anesthesia (RA + GA).

**Methods:**

Using the TriNetX database, patients undergoing primary TSA were identified and stratified into three groups based on anesthetic technique: GA, RA, and combined RA + GA. 1:1 Propensity score matching was performed to using the GA cohort as reference to control for baseline demographic and clinical covariates. Outcomes of interest included 90-day complication rates including UTI, venous thromboembolism (VTE), acute kidney injury (AKI), prosthetic joint infection (PJI), pneumonia, constipation, and revision TSA, emergency department (ED) visits and hospital readmission, Foley catheter insertion, postoperative opioid prescriptions and mean opioid number of prescriptions. Statistical significance was set at *p* < 0.05.

**Results:**

Comparing GA with RA, each cohort consisted of 28,564 patients. RA was associated with lower rates of UTI, AKI, constipation, pneumonia, fewer patients prescribed opioids and fewer opioid prescriptions per patient while being associated with increased healthcare utilization compared to GA alone. Comparing RA + GA with GA alone, 17,926 matched patients were included in each cohort. RA + GA was associated with lower rates of UTI and opioid prescriptions, and higher rates of healthcare utilization compared to GA alone.

**Conclusion:**

These findings suggest that while RA provides early analgesic and opioid-sparing benefits after TSA, it is associated with increased post-operative healthcare utilization. These findings help guide surgical decision making and highlight the need for standardized perioperative protocols to optimize postoperative pain control and manage rebound pain–related care utilization with RA techniques.

**Level of Evidence:**

III.

**Supplementary Information:**

The online version contains supplementary material available at https://doi.org/10.1007/s00590-026-04990-w.

## Introduction

Total shoulder arthroplasty is a highly effective procedure that produces large, durable improvements in pain and function [1, 2]. The number of patients undergoing total shoulder arthroplasty (TSA) has increased substantially in the past decade with a reported 212% increase in incidence from 2012 to 2022 and projections to continue rising over the next decade, with population-based studies demonstrating a marked increase in lifetime risk compared to prior decades [3–5]. Despite these excellent functional outcomes, TSA remains associated with meaningful perioperative morbidity and mortality. Reported 30-day complication rates range from 3.6 to 8%, with major morbidity occurring in 2–2.5% of cases and 30-day mortality of approximately 0.25% [6, 7].

General anesthesia (GA) remains the predominant anesthetic technique used for TSA, though regional anesthesia (RA) utilization is increasing. In a large analysis of 588 United States hospitals from 2010 to 2015, 79.1% of shoulder arthroplasty patients received GA alone, 17.8% received GA combined with RA (peripheral nerve block), and 3.1% received RA alone [8]. Similarly, a Nationwide Inpatient Sample analysis from 2007 to 2011 found that approximately 21% of TSA patients received RA in the form of upper-extremity peripheral nerve block in addition to GA [9]. The National Anesthesia Clinical Outcomes Registry (NACOR), which captures anesthesia-specific data, reported the highest rate of interscalene block use at 42.1%, with a significant year-over-year increase from 2010 to 2014 [10].

Moreover, prior studies evaluating anesthetic modality in TSA have reported inconsistent findings regarding perioperative outcomes, with some demonstrating reduced complication rates with RA and others showing no significant differences between anesthetic approaches. In a multi-hospital analysis of over 67,000 shoulder arthroplasty patients, GA alone, compared to GA plus RA, was associated with a 16% increase in all-cause infectious complications and 2.6-fold higher odds of pulmonary complications, as well as increased blood transfusions, intensive care unit transfers, and prolonged length of stay [8]. In contrast, another large study found that the addition of RA to GA was not associated with decreased the risk for complications following TSA [9]. The American Academy of Orthopaedic Surgeons/Major Extremity Trauma Research Consortium clinical practice guideline provides a strong recommendation for continuous RA over local anesthesia in TSA to reduce pain and opioid use in the first 24 hours after surgery [11]. Phrenic nerve palsy remains a significant consideration with interscalene blocks, particularly in patients with pre-existing pulmonary disease, and strategies to mitigate this include limiting local anesthetic volume and targeting more peripheral nerves such as the suprascapular and axillary nerves [12].

However, it remains unclear whether perioperative complication risk differs between GA and RA in TSA. Given this uncertainty, the projected increase in TSA volume as well as increase in RA use, it is important to understand anesthetic technique profiles in terms of outcomes and complications. As such, the present study aims to compare perioperative outcomes and complications across anesthetic techniques in patients undergoing TSA, including RA, GA, and combined regional and general (GA + RA) techniques. We hypothesize that RA, alone or in combination with GA, is associated with a lower incidence of perioperative complications and opioid need compared with GA alone.

## Methods

### Data source

We queried the TriNetX database using the Research Network, which compiles deidentified patient data from more than 100 healthcare organizations recording diagnoses, procedures, medications, lab values, and genomic data from over 100 million patients. As such, no Institutional Review Board approval was needed to conduct this study. Current Procedural Terminology (CPT), SNOMED (Systematized Nomenclature of Medicine) and ICD-10 (International Classification of Diseases, 10th Revision) codes were used to query the database. The query codes used for this study can be found in Supplementary Table 1.

### Inclusion/exclusion criteria and cohort formation

Data query was performed on May 14th, 2026. We queried for patients 18 years of age and older who were undergoing primary TSA for any indication. The included patients were then stratified into 3 cohorts based on the anesthesia modality received: GA alone, RA alone (single shot or continuous) and combined GA + RA. We followed-up both cohorts for up to 90 days after their TSA.

### 1:1 Propensity score matching

A 1:1 propensity matching was conducted using the GA cohort as reference to control for several relevant covariates similar to previously published methodology [13, 14]. These included demographics (age at surgery and sex) and comorbidities such as: body mass index (BMI; ≤25, 25–30, 30–40, and ≥ 40 kg/m²), hemoglobin A1c (≤ 5.6%, 5.6–6.5%, 6.5–7.5%, 7.5–8.5%, and ≥ 8.5%), insulin use, hypertension, disorders of lipoprotein metabolism and other lipidemias, inflammatory polyarthropathies, type 2 diabetes mellitus, ischemic heart disease, urinary tract infection, benign prostatic hyperplasia, liver disease, chronic obstructive pulmonary disease, acute cystitis, tobacco use, and chronic kidney disease stage (stages 1–5) and end-stage renal disease). The TriNetX platform uses a greedy nearest-neighbor algorithm, with adequate covariate balance assessed using a cutoff of standardized mean difference of < 0.1.

### Outcomes of interest

The primary outcomes assessed in this study were the 90-day incidence of complications including urinary tract infection (UTI), venous thromboembolism (VTE), acute kidney injury (AKI), prosthetic joint infection (PJI), pneumonia, constipation, and revision TSA, emergency department (ED) visits and hospital readmission, Foley catheter insertion, postoperative opioid prescriptions and mean opioid number of prescriptions.

### Statistical analysis

We used the built-in TriNetX system to perform statistical analysis. Risk percentages, odds ratios (ORs), 95% confidence intervals (95% CIs), and p-values were calculated for all outcomes. Categorical variables were assessed using the chi-squared test, while continuous variables were evaluated with Student’s t-tests. Statistical significance was set at *p* < 0.05.

## Results

The initial database query resulted in 108,506 patients, with 61,888 receiving GA only, 28,642 receiving RA only and 17,976 receiving combined RA + GA.

### RA versus GA

Comparing RA with GA prior to matching, the RA cohort had a higher proportion of patients with hypertensive diseases (66.35% vs. 54.47%, SMD = 0.245), disorders of lipoprotein metabolism and other lipidemias (58.86% vs. 44.32%, SMD = 0.294), inflammatory polyarthropathies (44.35% vs. 30.63%, SMD = 0.286), ischemic heart diseases (22.36% vs. 17.59%, SMD = 0.119), benign prostatic hyperplasia (12.60% vs. 8.45%, SMD = 0.136), diseases of liver (11.70% vs. 7.77%, SMD = 0.133) among other variables compared to the GA cohort. After matching, each cohort consisted of 28,564 patients with well-balanced baseline characteristics (Table 1).

**Table 1:** Baseline cohort characteristics before and after propensity score matching comparing the RA with the GA cohort

Characteristic name	Before matching			After matching		
	RA only (*n* = 28,642)	GA only (*n* = 61,888)	Standardized mean difference	RA only (*n* = 28,564)	GA only (*n* = 28,564)	Standardized mean difference
Age at Surgery (mean +/− standard deviation)	69.02 +/− 9.22	69.11 +/− 9.86	0.009	69.02 +/− 9.22	68.79 +/− 9.51	0.025
Female	54.40%	56.85%	0.049	54.46%	54.29%	0.003
Male	45.47%	43.01%	0.049	45.41%	45.56%	0.003
Hypertensive diseases	66.35%	54.47%	**0.245**	66.27%	66.80%	0.011
Disorders of lipoprotein metabolism and other lipidemias	58.86%	44.32%	**0.294**	58.76%	58.41%	0.007
Inflammatory polyarthropathies	44.35%	30.63%	**0.286**	44.21%	43.45%	0.015
Type 2 diabetes mellitus	23.67%	20.56%	0.075	23.64%	23.37%	0.007
Ischemic heart diseases	22.36%	17.59%	**0.119**	22.31%	21.76%	0.013
Urinary tract infection, site not specified	14.44%	11.42%	0.090	14.40%	13.62%	0.022
Benign prostatic hyperplasia	12.60%	8.45%	**0.136**	12.50%	11.86%	0.020
Diseases of liver	11.70%	7.77%	**0.133**	11.63%	10.92%	0.023
chronic obstructive pulmonary disease	11.05%	9.56%	0.049	11.04%	10.83%	0.007
Acute cystitis	5.96%	3.88%	0.096	5.94%	5.54%	0.017
Tobacco use	4.78%	3.21%	0.080	4.76%	4.62%	0.006
Insulin use	17.04%	15.35%	0.046	17.01%	16.50%	0.014
Chronic kidney disease, stage 1	0.54%	0.38%	0.024	0.54%	0.42%	0.017
Chronic kidney disease, stage 2 (mild)	2.42%	1.62%	0.057	2.41%	2.31%	0.006
Chronic kidney disease, stage 3 (moderate)	8.32%	6.36%	0.075	8.28%	7.86%	0.015
Chronic kidney disease, stage 4 (severe)	1.08%	1.18%	0.010	1.08%	1.01%	0.007
Chronic kidney disease, stage 5	0.28%	0.36%	0.013	0.28%	0.25%	0.007
End stage renal disease	0.68%	0.80%	0.014	0.68%	0.62%	0.008
Body mass index						
At most 25 kg/m^2^	28.82%	22.17%	**0.153**	28.69%	29.04%	0.008
25–30 kg/m^2^	48.12%	38.48%	**0.196**	48.01%	49.41%	0.028
30–40 kg/m^2^	51.57%	41.45%	**0.204**	51.48%	52.32%	0.017
At least 40 kg/m^2^	16.14%	12.35%	**0.108**	16.09%	15.94%	0.004
Hemoglobin A1c						
At most 5.6%	25.98%	15.42%	**0.263**	25.80%	24.55%	0.029
5.6–6.5%	33.20%	22.79%	**0.233**	33.09%	32.64%	0.010
6.5–7.5%	12.49%	9.73%	0.088	12.47%	12.25%	0.007
7.5–8.5%	6.46%	4.86%	0.069	6.44%	6.27%	0.007
At least 8.5%	4.41%	3.50%	0.047	4.40%	4.31%	0.004

RA, Regional anesthesia; GA, General anesthesia. Bold values indicate inadequate covariate balance (standardized mean difference > 0.1)

At 90 days postoperatively, RA was associated with lower rates of UTI (2.36% vs. 2.89%, *p* < 0.001), AKI (1.56% vs. 1.85%, *p* = 0.007), constipation (2.87% vs. 3.17%, *p* = 0.040), pneumonia (0.88% vs. 1.18%, *p* < 0.001), fewer patients prescribed opioids (70.89% vs. 73.36%, *p* < 0.001), and fewer mean number of opioid prescriptions per patient (2.41 ± 2.66 vs. 2.57 ± 2.92, *p* < 0.001). By contrast, RA was associated with higher rates of ED visits (7.85% vs. 6.91%, *p* < 0.001) and readmissions (5.94% vs. 5.16%, *p* < 0.001). Rates of Foley catheter insertion (0.27% vs. 0.34%, *p* = 0.148), VTE (1.20% vs. 1.19%, *p* = 0.878), PJI (1.33% vs. 1.27%, *p* = 0.507), and revision TSA (1.19% vs. 1.13%, *p* = 0.482) were similar between groups (Table 2).

**Table 2:** 90-day outcomes after propensity score matching comparing the RA with the GA cohort

Complication	RA only (*n* = 28,564)	GA only (*n* = 28,564)	OR (95% CI)	*p*-value
Foley catheter insertion	0.27%	0.34%	0.80 (0.59–1.08)	0.148
UTI	2.36%	2.89%	0.81 (0.73–0.90)	**< 0.001**
VTE	1.20%	1.19%	1.01 (0.87–1.18)	0.878
AKI	1.56%	1.85%	0.84 (0.74–0.95)	**0.007**
PJI	1.33%	1.27%	1.05 (0.91–1.21)	0.507
Revision TSA	1.19%	1.13%	1.06 (0.91–1.23)	0.482
ED visit	7.85%	6.91%	1.15 (1.08–1.22)	**< 0.001**
Readmission	5.94%	5.16%	1.16 (1.08–1.25)	**< 0.001**
Constipation	2.87%	3.17%	0.90 (0.82–0.99)	**0.040**
Pneumonia	0.88%	1.18%	0.75 (0.64–0.88)	**< 0.001**
Opioids	70.89%	73.36%	0.88 (0.85–0.92)	**< 0.001**
Number of opioid prescription (mean +/− standard deviation)	2.41 +/− 2.66	2.57 +/− 2.92	–	**< 0.001**

GA, General anesthesia; RA, Regional anesthesia; OR, Odds ratio; CI, Confidence interval; UTI, Urinary tract infection; AKI, Acute kidney injury; PJI, Prosthetic joint infection; VTE, Venous thromboembolism; TSA, Total shoulder arthroplasty; ED, Emergency department; bold values indicate statistical significance (*p* < 0.05)

### RA + GA versus GA

Comparing RA + GA with GA alone, the RA + GA cohort had a higher proportion of patients with hypertensive diseases (66.39% vs. 54.47%, SMD = 0.246), disorders of lipoprotein metabolism and other lipidemias (58.14% vs. 44.32%, SMD = 0.279), inflammatory polyarthropathies (45.51% vs. 30.63%, SMD = 0.310), ischemic heart diseases (24.18% vs. 17.59%, SMD = 0.163), benign prostatic hyperplasia (11.97% vs. 8.45%, SMD = 0.116), diseases of liver (11.00% vs. 7.77%, SMD = 0.111), tobacco use (6.28% vs. 3.21%, SMD = 0.145) among other variables, when compared to the GA cohort. After matching, 17,926 matched patients were included in each cohort with adequate balance of baseline covariates (Table 3).

**Table 3:** Baseline cohort characteristics before and after propensity score matching comparing the RA + GA with the GA cohort

Characteristic name	Before matching			After matching		
	RA + GA (*n* = 17,976)	GA only (*n* = 61,888)	Standardized mean difference	RA + GA (*n* = 17,926)	GA only (*n* = 17,926)	Standardized mean difference
Age at Surgery (mean +/− standard deviation)	68.56 +/− 9.38	69.11 +/− 9.86	0.057	68.56 +/− 9.38	68.40 +/− 9.65	0.017
Female	55.67%	56.85%	0.024	55.69%	55.91%	0.004
Male	44.33%	43.01%	0.027	44.31%	44.00%	0.006
Hypertensive diseases	66.39%	54.47%	**0.246**	66.30%	67.60%	0.028
Disorders of lipoprotein metabolism and other lipidemias	58.14%	44.32%	**0.279**	58.02%	58.12%	0.002
Inflammatory polyarthropathies	45.51%	30.63%	**0.310**	45.37%	46.10%	0.015
Type 2 diabetes mellitus	24.72%	20.56%	0.100	24.61%	24.32%	0.007
Ischemic heart diseases	24.18%	17.59%	**0.163**	24.05%	23.72%	0.008
Urinary tract infection, site not specified	14.23%	11.42%	0.084	14.20%	14.00%	0.006
Chronic obstructive pulmonary disease	12.56%	9.56%	0.096	12.52%	12.32%	0.006
Benign prostatic hyperplasia	11.97%	8.45%	**0.116**	11.89%	11.43%	0.015
Diseases of liver	11.00%	7.77%	**0.111**	10.93%	10.67%	0.008
Insulin use	14.81%	15.35%	0.015	14.82%	14.11%	0.020
Tobacco use	6.28%	3.21%	**0.145**	6.17%	6.00%	0.007
Acute cystitis	5.73%	3.88%	0.087	5.71%	5.63%	0.003
Chronic kidney disease, stage 1	0.52%	0.38%	0.021	0.51%	0.59%	0.010
Chronic kidney disease, stage 2 (mild)	2.24%	1.62%	0.045	2.24%	2.22%	0.002
Chronic kidney disease, stage 3 (moderate)	9.12%	6.36%	**0.103**	9.09%	8.43%	0.023
Chronic kidney disease, stage 4 (severe)	1.27%	1.18%	0.008	1.27%	1.22%	0.004
Chronic kidney disease, stage 5	0.33%	0.36%	0.005	0.33%	0.24%	0.017
End stage renal disease	0.61%	0.80%	0.022	0.61%	0.54%	0.010
Body mass index						
At most 25 kg/m^2^	20.22%	22.17%	0.048	20.26%	21.08%	0.020
25–30 kg/m^2^	38.57%	38.48%	0.002	38.62%	39.62%	0.020
30–40 kg/m^2^	44.79%	41.45%	0.067	44.81%	46.43%	0.033
At least 40 kg/m^2^	14.47%	12.35%	0.062	14.46%	14.82%	0.010
Hemoglobin A1c						
At most 5.6%	24.85%	15.42%	**0.237**	24.69%	24.09%	0.014
5.6–6.5%	32.30%	22.79%	**0.214**	32.13%	32.31%	0.004
6.5–7.5%	14.10%	9.73%	**0.135**	13.97%	13.72%	0.007
7.5–8.5%	7.62%	4.86%	**0.114**	7.53%	7.39%	0.005
At least 8.5%	5.54%	3.50%	0.098	5.45%	5.31%	0.006

RA+GA= combined regional and general anesthesia, GA=general anesthesia.Bold values indicate inadequate covariate balance (standardized mean difference >0.1)

At 90 days postoperatively, RA + GA was associated with lower rates of UTI (2.70% vs. 3.11%, *p* = 0.020) and fewer patients prescribed opioids (72.01% vs. 73.46%, *p* = 0.002). RA + GA was associated with higher rates of ED visits (9.95% vs. 7.03%, *p* < 0.001) and readmissions (6.01% vs. 4.94%, *p* < 0.001). Rates of Foley catheter insertion (0.32% vs. 0.36%, *p* = 0.468), VTE (1.07% vs. 1.26%, *p* = 0.085), AKI (1.70% vs. 1.95%, *p* = 0.076), PJI (1.16% vs. 1.18%, *p* = 0.844), revision TSA (1.37% vs. 1.19%, *p* = 0.145), constipation (2.91% vs. 3.23%, *p* = 0.081), pneumonia (1.10% vs. 1.28%, *p* = 0.108), and mean number of opioid prescriptions (2.63 ± 2.70 vs. 2.59 ± 2.91, *p* = 0.194) were similar between groups (Table 4).

**Table 4:** 90-day outcomes after propensity score matching comparing the RA + GA with the GA cohort

Complications	RA + GA (*n* = 17,926)	GA only (*n* = 17,926)	OR (95% CI)	*p*-value
Foley catheter insertion	0.32%	0.36%	0.88 (0.61–1.25)	0.468
UTI	2.70%	3.11%	0.86 (0.76–0.98)	**0.020**
VTE	1.07%	1.26%	0.84 (0.70–1.02)	0.085
AKI	1.70%	1.95%	0.87 (0.74–1.02)	0.076
PJI	1.16%	1.18%	0.98 (0.81–1.19)	0.844
Revision TSA	1.37%	1.19%	1.15 (0.95–1.38)	0.145
ED visit	9.95%	7.03%	1.46 (1.36–1.58)	**< 0.001**
Readmission	6.01%	4.94%	1.23 (1.12–1.35)	**< 0.001**
Constipation	2.91%	3.23%	0.90 (0.80–1.01)	0.081
Pneumonia	1.10%	1.28%	0.86 (0.71–1.04)	0.108
Opioids	72.01%	73.46%	0.93 (0.89–0.97)	**0.002**
Number of opioid prescription (mean +/− standard deviation)	2.63 +/− 2.70	2.59 +/− 2.91	–	0.194

GA, General anesthesia; RA, Regional anesthesia; OR, Odds ratio; CI, Confidence interval; UTI, Urinary tract infection; AKI, Acute kidney injury; PJI, Prosthetic joint infection; VTE, Venous thromboembolism; TSA, Total shoulder arthroplasty; ED, Emergency department; bold values indicate statistical significance (*p* < 0.05)

## Discussion

As the volume of patients undergoing TSA continues to rise, identifying optimal anesthetic techniques has become increasingly relevant. Therefore, this retrospective cohort study was performed to analyze the use of RA, GA and RA + GA in TSA procedures. Our study found that patients receiving RA, when compared with those receiving GA alone, had lower rates of UTIs, pneumonia, constipation, and opioid prescriptions but higher rates of healthcare utilization (ED visits and readmission). These findings provide additional context to the existing literature on RA, suggesting that reductions in perioperative complications may not necessarily translate into lower postoperative healthcare utilization.

The results of the present study showing decreased opioid prescriptions post-TSA with RA use are consistent with the findings of prior research. RA has been associated with better analgesia and decreased opioid prescriptions after joint arthroplasty. Kolade et al., in a study of 622 patients undergoing TSA, found that GA was associated with higher opioid prescriptions compared to RA [15]. Similarly, McCormick et al. compared 4,748 patients receiving RA following total hip arthroplasty with 23,740 matched controls and reported that RA was associated with a 30.2% lower daily opioid requirement (38.6 vs. 55.3 morphine milligram equivalents/day, *p* < 0.001) [16]. Along the same lines, comparing RA with GA, Ledford et al. also found that patients receiving RA had a lower discharge pain score as well as lower inpatient opioid need among patients with chronic opioid use undergoing primary total knee arthroplasty [17]. This is particularly important especially within the context of the ongoing opioid crisis, where between 1999 and 2021, there were approximately 280,000 deaths attributed to opioid addiction, with orthopedic surgeons holding the third highest opioid prescription rates [18], and patients having high numbers of remaining pills after elective orthopedic procedures [19].

In addition, the present study also found that RA was associated with lower rates of UTIs, pneumonia, constipation, and opioid prescriptions, when compared with GA alone. These findings are consistent with prior literature in shoulder arthroplasty. Herrick et al., demonstrated that GA alone was associated with a 16% increase in infectious complications and 2.6-fold higher odds of pulmonary complications compared with RA + GA in shoulder arthroplasty patients [12]. Stundner et al., in a nationwide analysis of 17,157 TSA patients, found that the addition of RA did not increase complication rates or resource utilization [8]. Illescas et al. evaluated over 3 million total hip and knee arthroplasty patients and reported that RA was associated with lower risks of pneumonia, infections, acute renal failure, cardiac complications, and ICU admissions when compared to GA alone [20]. Similar benefits of RA have been demonstrated in other hip and knee arthroplasty analyses, with large registry studies showing reductions in infections, pulmonary complications, and length of stay [21, 22].

The decreased rates of pneumonia in our study as well as others may be partly attributable to the avoidance of endotracheal intubation with RA, as intubation has been associated with impaired mucociliary clearance, bypassed upper airway defenses, and increased risk of bacterial entry into the lower respiratory tract [23, 24]. The decreased UTI rates within the RA cohort could be secondary to reduced opioid consumption, as opioids can cause urinary retention through inducing detrusor relaxation, impairing bladder sensation, and increasing sphincter tone [25, 26]. Postoperative urinary retention, which affects up to 43% of orthopedic patients, frequently necessitates catheterization which is a risk factor for catheter-associated UTI [27]. Additionally, GA may secondarily contribute to urinary retention through the smooth muscle relaxation effect of neuromuscular blockade reversal agents [28]. Similarly, the lower constipation rates observed with RA are likely attributable to reduced opioid exposure, as opioids can cause decreased intestinal motility, reduced mucosal secretions, and increased sphincter tone [29]. In a study of over 788,000 lower extremity arthroplasty patients, opioid-induced constipation was associated with longer hospital stays, higher costs, and increased 30-day readmissions and ED visits [30]. Cozowicz et al. further demonstrated a dose-response relationship in over 1 million arthroplasty patients, with higher opioid prescription associated with 23% increased risk of urinary complications and over 15% increased risk of gastrointestinal complications [31].

Interestingly, our study found that, despite lower rates of post-operative complications, RA is associated with higher rates of ED visits and hospital readmissions. These findings present a seemingly paradoxical relationship of decreased complications but increased post-operative healthcare utilization. One potential explanation to these observations is the rebound pain effect associated with resolution of peripheral nerve blocks with RA. Peripheral nerve blocks with RA have been shown to wear off, with their peak pain occurring at 24 hours after injection [32, 33]. Patients can experience an abrupt transition from near complete analgesia to high rates of pain [34]. With the temporal relationship of increased pain associated with RAs, it is possible that that the late-onset of severe pain led patients to seek out medical care. Additionally, prior literature has demonstrated that continuous interscalene nerve block catheters for shoulder surgery can cause pulmonary complications, like hypoxemia from phrenic nerve blockade, leading to unanticipated need for medical attention and significant associated costs [35]. Alternatively, patients receiving RA may be released sooner after TSA procedures and subsequently rely on the ED for post-operative care. Strategies including multimodal analgesia, timely initiation of oral analgesics before block resolution, and standardized postoperative pain pathways may help mitigate the effects of rebound pain. Additionally, thorough preoperative patient counseling regarding the expected duration of regional anesthesia and the potential for transient rebound pain may reduce anxiety-driven healthcare encounters and improve postoperative satisfaction. Further studies however are needed to confirm this relationship between rebound pain and healthcare utilization and whether the aforementioned approaches can effectively mitigate it.

This study is not without limitations. As a retrospective database study, our findings are limited by the variability and accuracy in coding across hospitals and providers and completeness of documentation. Clinical events occurring outside contributing healthcare systems or information not captured within structured electronic health records fields may also be unavailable. Additional imputation of missing clinical data could not be performed in this study since we only accessed the compiled deidentified data as provided by the database. Similarly, our cohorts, propensity matching, and outcome variables relied on ICD-10 and CPT codes and therefore are subject to potential misclassification. Another limitation is that the type of anesthetics used such as the specific block type, medication, dose, or separating single-shot from continuous techniques couldn’t be reliably reported. Furthermore, patients undergoing anatomic and reverse shoulder arthroplasty were grouped together. Moreover, we cannot confirm the reasons for emergency department or hospital visits. Additionally, hospital length of stay was not available for analysis as in inherent limitation to the database and may also influence the overall interpretation of resource utilization. Despite propensity matching for chronic diseases to mitigate confounding factors, residual confounding factors like surgeon preference, hospital-specific protocols, socioeconomic status, postoperative support systems, anesthesia selection criteria as well as the indications for TSA, and intraoperative factors such as surgical duration might remain. Despite these limitations, this study draws data from an aggregate electronic health record that includes diverse healthcare organizations and validates prior research in a larger and more heterogeneous population across geographic regions, demonstrating the reproducibility of RA benefits across populations. Future studies should consider measuring rebound pain intensity in conjunction with RA and RA + GA anesthetics, evaluating the cost-effectiveness for RA considering the decreased complications, post-operative healthcare utilization, as well as investigating potential analgesic options and protocols to combat rebound pain.

## Conclusion

TSA performed under RA was associated with lower rates of postoperative complications while also being associated with increased post-operative healthcare utilization compared with GA. Overall, these findings suggest a tradeoff between improved perioperative safety outcomes and increased downstream resource use with the use of RA in patients undergoing TSA. Importantly, the opioid-sparing effects observed with RA are particularly relevant in the context of ongoing efforts to reduce opioid exposure within orthopedic surgery and represent a meaningful clinical advantage. While these findings support the continued use of RA in appropriately selected patients, further prospective studies are needed to better characterize the drivers of increased healthcare utilization and to evaluate the overall effectiveness of RA, balancing its clinical benefits against potential increases in postoperative resource utilization.

## Supplementary Information

Below is the link to the electronic supplementary material.

Supplementary Material 1

## Data Availability

The data supporting the findings of this study were obtained from the TriNetX research network (TriNetX, Cambridge, MA, USA), a federated network of de-identified electronic health records from participating healthcare organizations. Restrictions apply to the availability of these data, which were used under license for the current study and are therefore not publicly available. The data are, however, accessible to authorized users of the TriNetX platform upon reasonable request and with appropriate institutional access.
