# Supplementary material for: Anesthetic technique is associated with complication risk and healthcare utilization following total shoulder arthroplasty
Source: Eur J Orthop Surg Traumatol. 2026 Oct 21;36(1):414. doi: 10.1007/s00590-026-04990-w (PMC13687372; doi:10.1007/s00590-026-04990-w)
Supplement: Supplementary file 1 [file 590_2026_4990_MOESM1_ESM.docx]

Supplementary Table 1: Summary of the codes used to query the database

| Variable | Code |
| --- | --- |
| Shoulder arthroplasty | CPT 23472, ICD-10-PCS 0RRJ008, 0RRJ00Z, 0RRJ0JZ, 0RRK008, 0RRK00Z, 0RRK0JZ |
| General Anesthesia | CPT 01638, 01630, 01610 |
| Regional Anesthesia | CPT 64415, 64418, 64417, 64416 |
| Foley catheter insertion | CPT 51702, 51703 |
| UTI | ICD-10: N39.0, N30.0 |
| VTE | ICD-10: I82.4, I26 |
| AKI | ICD-10: N17 |
| PJI | ICD-10: T84.50, T84.59 |
| Revision TSA | CPT 1021145, ICD-10-PCS 0RWJ, 0RWK |
| Readmission | CPT 99221, 99222, 99223 |
| Pneumonia | ICD-10: J13-18 |
| Constipation | ICD-10: K59.0 |
| Opioids | VA: CN101 |
| ED visit | CPT 1013711 |
| CPT = Current Procedural Terminology codes for medical procedures; ICD-10 = International Classification of Diseases, 10th Revision diagnostic codes; UTI = urinary tract infection; VTE = venous thromboembolism (deep vein thrombosis or pulmonary embolism); AKI = acute kidney injury; PJI = periprosthetic joint infection; ED = emergency department visits; TSA = total shoulder arthroplasty. | |
